# Notes and News

**Published:** 1887-09-10

**Authors:** 


### NOTES AND NEWS.

COLLECTED AND COMMUNICATED.

HER Majesty the Queen appears to have declined to lay the first stone of the Aberdeen Royal Infirmary extension. The loyal people of Aberdeen seem decidedly hurt, and, as is not unusual in Scotland, have expressed their feelings freely.

THE death is announced of Lady Millicent Bence-Jones, which took place at 64, Great Cumberland Place, Hyde Park. Her ladyship, who was the daughter of the second Earl of Gosford, married, in 1842, Mr. Bence-Jones, for some time consulting surgeon to St. George's Hospital, and secretary to the Royal Institution.

AS Lady Millbank and her daughter were driving tandem through Ipswich the leader bolted, and the vehicle, coming in contact with a drag, was overturned, both ladies being injured, Miss Millbank so seriously that she has since died.

FELIXSTOWE has held a bazaar, an annual event, in aid of the Suffolk Convalescent Home. The sale was highly successful, and realised £140 14s. 11d. ; more than £40 in excess of last year's total.

EIGHT ounces of laudanum were given to a racehorse at Londonderry by some scoundrel who was interested in preventing its running. The animal seems to have quite recovered from the effects of the dose, though not in time to take part in the race.

A NEW wing has been added to the Bootle Borough Hospital, at the cost of £8,930 ; the architect being Mr. C. O. Elison, M.S.A., of Liverpool.

624 patients were under treatment at the Royal Ear Hospital, Frith Street, Soho Square, during the month of August.

IT has been decided at the Devon and Exeter Hospital to appoint a second resident medical officer in addition to the house-surgeon, and to give him an honorarium of £40 a year in addition to board and lodging.

THE North Lonsdale Hospital, the permanent premises of which were opened in May last, has just held its annual meeting. The report of the secretary states that in consequence of the continued depression in trade there has been some falling off in income. In other respects the report is satisfactory.

MR. WELLER, the dispenser at the Brighton Infirmary, has been compelled to retire in consequence of ill-health, after a connection of thirty-one years with the institution. It is gratifying to note that the governors have conferred upon Mr. Weller a pension of £26 per annum for the remainder of his life.

A HOUSE-TO-HOUSE collection is made annually at Lincoln on behalf of the hospital. That for the present year will be taken in conjunction with the Hospital Saturday collection on the 24th of September. We shall be glad if some reader at Lincoln will inform us of the actual amount received by those who go from house to house on that date.

THE town of Batley, near Leeds, gives an annual Sunday concert in the open air, in aid of the funds of its Cottage Hospital. This year the concert was held in the cricket-field in the finest of weather. There was a full band and a chorus of 400 performers. Needless to say, the attendance was large, and the performance greatly enjoyed by the music-loving population of the town and Riding.

THE liberal promises made on behalf of the funds of the new Children's Ward at the Newport Infirmary are being realised rapidly and satisfactorily. We record this rather unusual circumstance in order that other promisers to other hospitals may be reminded that promises made are expected to be fulfilled within a reasonable period ; at any rate some time before the end of the century.

THE Glasgow Medical Charities Committee, which a few months ago was full of valour and resolution, seems to have collapsed entirely, and the daily papers of that city are laughing at the somewhat ridiculous figure the Committee is cutting. Is there a split in the camp ? Scotsmen are famous fighters when they can agree about the cause of battle ; but for "hair splitting" and disputation about trifles commend us to a typical Scot.

The following vacancies are announced this week, the amount of the salary being placed in each case after the name of the institution :—

**Resident Medical Officers.**

County Down Infirmary. Registrar, Dispenser, and Surgical Assistant.—£63.
Devon and Exeter Hospital.—£120.
Manchester Royal Infirmary. House Surgeon.—£150.
Royal Berks Hospital, Reading. Assistant House Surgeon.—No Salary.
St. Luke's Hospital. Resident Clinical Assistant.

**Non-resident Medical Officers.**

Bristol Royal Infirmary. Obstetric Physician.
Evelina Hospital (Children's). Out-patients' Surgeon.
Great Northern Central Hospital. Obstetric Physician.
Sheffield General Infirmary. Two Honorary Assistant Physicians.
Sheffield General Infirmary. Two Honorary Assistant Surgeons.

**Matron.**

Malvern College Sanatorium. £112 10s.

**Trained Nurses.**

Burlington House, Boundary Road, N.W. Several Trained Nurses. Apply to Lady Superintendent.
Eastern Fever Hospital, The Grove, Homerton, E.—£30 to £36. Assistant, £22 to £26.
Mental, Attendant and Companion. Clayton House, Westcroft Road, South Hampstead.
South-Western Hospital, Stockwell. Day and Night Nurses, £30 to £36. Assistant, £22 to £26.

---

Hospital patients and poor people in the country should now be able to enjoy a little change of diet. The reports of the partridge shooting which commenced on the first are favourable. Coveys are large and the birds strong aud plump. Is it necessary to remind sportsmen that game is most acceptable at hospitals, and that it cannot generally be obtained except when sent as a free gift?

---

Miss Hilbery has been appointed matron of the Royal Sea-bathing Infirmary, Margate, in the place of Miss Collier who has recently resigned, after holding office for twenty years. Miss Hilbery was trained at St. Mary's Hospital, Paddington ; where, in addition to the ordinary duties, she has had charge of the casualty department, and has acted as night superintendent. The Sea-bathing Infirmary, which has 220 beds, has reason to congratulate itself on this recent addition to the resident staff. More than forty candidates applied for the post.

---

When a diminished income is counterbalanced by a lessened expenditure, on Mr. Micawber's philosophy, happiness is possible. At the annual meeting of the Governors of the Stratford-upon-Avon Infirmary, held on Monday, the hon. sec., Mr. C. A. Garnett, was able to report that in spite of lessened resources all claims occurring during the year had been met without borrowing. The work of the year has been done smoothly and satisfactorily, and a due meed of praise was awarded to the honorary staff and the matron and nurses. The chairman, Sir Arthur Hodgson, K.C.M.G., attributed the successful management of the finances to the earnestness and skilful management of the honorary secretary, Mr. C. A. Garnett, without whose generous aid the report could not have been so satisfactory. The Marquis of Hertford was elected president in the place of Sir Robert Hamilton, deceased, and Mr. Garnett was re-appointed hon. sec. for the ensuing year.

"A cheap trip" is the form of diversion favoured by the Infirmary Saturday Committee at Derby this year. The members with their wives and friends found their way to Ashford-on-the-Water, near Bakewell. An excellent tea was provided at the Devonshire Arms Hotel, under the stimulus of which Mr. Woodward, who presided, and various speakers, urged the claims of the infirmary, and testified to the noble work it was doing.

---

The annual sermons in aid of the Isle of Wight Infirmary were preached at St. Thomas's Church, Newport, on Sunday. The morning preacher was the Archdeacon of the Isle of Wight, and the evening service was taken by the Rev. C. S. Nichol. The Archdeacon stated that in 1886 the total amount collected in all the places of worship in Newport only reached £46 ; in 1885 the sum of £65 15s. 6d.; whilst in 1884, £63 17s. 3d. was the total. It therefore appeared, said the Archdeacon, that they had been going from bad to worse.

---

At Leamington the annual collections in aid of the Warneford Hospital were made on Saturday. The weather was very unfavourable, and the returns from the street stands suffered in consequence. Messrs. Urwin, of Fern Nursery, Bedford Street, F. Perkins, Regent Street, and E. Perkins, Avenue Road Nursery, lent beautiful flowers for stand decorations, Mr. Urwin paying special attention to the stand near the pump rooms. The committee are sanguine that the total amount raised will, in spite of the weather, be equal to that realised last year.

---

"Necessity is the mother of invention." A correspondent of the *Hastings Observer* is much concerned to find that the new hospital, which is greatly admired, is beginning to feel the pinch of straitened means. He suggests that it would be a good idea to turn the building into an "exhibition" for a week or two. He thinks that if a public announcement were made to the effect that the building would be "on show" for a week or longer, and that a small charge would be made, large numbers of people would visit it with the double object of gratifying their curiosity and giving it a helping hand. The suggestion is well worth a consideration.

---

The weekly workshop collection on behalf of the London hospitals, which is conducted by the Hospital Saturday Fund, was brought to a close last Saturday No fewer than 24,000 business establishments have been appealed to this year,—a largely increased number as compared with former years. The managers of the Fund display a constantly increasing sense of the importance of their work and of its possibilities of almost unlimited development. We are glad to see that they are stirring to popularise the movement by public demonstrations and open-air *fêtes*. If the hospitals are to be maintained in efficiency it can only be by enlisting the sympathy and active help of the whole population. What is impossible to a thousand may be perfectly easy to a million.

The Southwark Teachers' Association unanimously resolved at their annual meeting to raise a fund to contribute towards the deficit at Guy's Hospital. About thirty-five schools have taken part in the effort, and the very satisfactory total of nearly £140 has been raised. The children and parents, as well as the teachers, have joined in the movement.

---

Collecting dogs are not very common, and when they are employed are sometimes very successful. The Friendly Societies of the western district gave one of their demonstrations recently in aid of the Acton Cottage Hospital and the West London Hospital, and perhaps their most successful collector was a friendly dog, who, with sundry ingenious contrivances strapped to his back, proved himself to be a valuable receiving office. The amount given to this canine collector alone was £3 15*s.* 6*d.* The total sum received in the course of the demonstration was £68 18*s.* 6*d.*

---

The London Temperance Hospital has spent on its buildings, from first to last, about £50,000, all of which has been paid except £6,500. It is claimed on behalf of the hospital that it has done very much to break down the ancient faith in the curative value of alcohol. This claim may be readily admitted, but considering that the hospital has been in existence for thirteen years, it does not say much for the generosity of total abstainers that it should be still in debt to the amount of nearly £7,000, and that want of funds prevents it from receiving many patients who would otherwise be glad to place themselves under its care.

---

The Directors of the Great Northern Railway Company have voted a donation of one hundred pounds towards the building-fund of the Great Northern Central Hospital. The Islington Jubilee Fund, in aid of the same object, is said to have reached between three and four thousand pounds, and the ladies' fund nearly one thousand. The central wing of the new hospital, containing sixty beds in their principal wards, and several private wards for paying patients, is now roofed in, and will be ready for occupation in a few weeks. The general architectural features, and the sanitary details of that portion of the hospital which is so near completion, are in many respects admirable, and a visit to Holloway Road will be amply repaid. The spectator cannot help feeling a slight shock on first seeing the mutilated and even decapitated appearance of the structure. The funds collected only allow of the erection of one wing and the out-patients' department; and thus the impression is produced of a trunk with only one leg and minus a head. If the committee are really anxious to finish the building they should give a reception on the other side of the road to all people who have ever been known to be generous to hospitals. During the reception the unfinished and forlorn spectacle would plead so eloquently for aid, that nobody would be able to go away without giving a handsome donation on the spot. Seriously, the building looks most piteous in its present plight, and the managers must make some kind of effort to complete it. About twenty thousand pounds would give North London in the new hospital a hundred and twenty beds, in addition to wards for paying patients. We venture to say a word to our readers on behalf of one of the most necessary and deserving medical charities in London. The Great Northern Central is practically the only hospital available for a million people.

---

The fever epidemic continues to extend, but not at an alarming rate. Up to date the number of scarlet fever cases in the hospitals of the Metropolitan Asylums Board approaches a thousand, which figures are by no means extraordinary. It must be understood that there are many cases scattered over the metropolis and suburbs which do not come within the cognisance of the Board, and which, in consequence of the want of a Compulsory Registration Act, are not reported. But making due allowance for these unreported cases, it cannot be said that there is any special reason for public alarm. The people generally seem to be disposed to rely upon the authorities and the medical profession, and manifest a confidence in their intelligence and capacity which is as gratifying as it is undoubtedly well grounded.

---

Mr. F. R. Russell, writing to *The Times* concerning the present epidemic of scarlet fever, says:—"Your article of to-day places in a very strong light the almost superstitious reluctance of the human mind to apply the resources of science to the destruction of the greatest evils which prey upon the health and happiness of mankind. The annihilation of small-pox by vaccination, of typhoid fever by clean surroundings, and of hydrophobia by the consistent supervision and control of dogs, as in Sweden, Germany, and Australia, fail to impress the majority of the public with the magnitude and certainty of the advantage gained, else the sole inconveniences entailed by well-devised precautions would not weigh for a single moment against the immense and intense suffering permitted by *laissez-faire* inactivity. Is there any reason why a war of extermination should not be carried on in England against typhoid and scarlet fever as successfully as was once undertaken against the wolves, or, if extermination be impossible, is not a great reduction of those diseases—which is possible—worth a great national effort? You stated that scarlet fever has in the last thirty years destroyed the lives of more than half a million of persons, and occasioned an expense of about £6,000,000. In that time about five million persons must have suffered from this fever, and many must have been disabled for life. Why are vast sums given to hospitals and almost nothing to the means by which their inmates might have been saved from their misery and loss? There must be many who know that the worst infectious disorders can be suppressed by scientific treatment, and in default of any national or State action would be glad to contribute towards the full investigation of the cause and spread of this fever which is now disseminated in London, and must be a source of much anxiety in a vast population which has not yet adopted compulsory registration of infectious cases. If any such investiga- is started and well supported, I for one should be very glad to contribute £50 a year for three years to so great and promising a purpose."

